# The Arab Community in Israel Coping with Intellectual and Developmental Disability

**DOI:** 10.1100/tsw.2004.31

**Published:** 2004-05-11

**Authors:** Isack Kandel, Mohammed Morad, Gideon Vardi, Joseph Press, Joav Merrick

**Affiliations:** ^1^ Faculty of Social Science, Department of Behavioral Sciences, Academic College of Judea and Samaria, Ariel, DN Ephraim 44837, Israel; ^2^ Division of Community Health, Ben Gurion University, Beer-Sheva, Israel; ^3^ Zusman Child Development Center, Division of Pediatrics, Ben Gurion University, Beer-Sheva, Israel; ^4^ Pediatric Emergency Department, Division of Pediatrics, Soroka University Medical Center, Ben Gurion University, Beer-Sheva, Israel; ^5^ National Institute of Child Health and Human Development, Office of the Medical Director, Division for Mental Retardation, Ministry of Social Affairs, Jerusalem and Division of Pediatrics, Ben Gurion University, Beer-Sheva, Israel

**Keywords:** mental retardation, developmental disability, intellectual disability, human development, child health, public health, Israel

## Abstract

The Arab family in Israel is still embedded in the traditional society with extended family support systems, but we see a population in transition influenced by the surrounding society. This paper looks at the different religious attitudes toward the exceptional people in our society (i.e., the family reaction to a child born with intellectual or developmental disability), reviews recent studies on the Arab and Bedouin families in Israel, and presents data on the Arab population in residential care centers.Today, out of 57 residential care centers in Israel for persons with intellectual disability, 13 (22.8%) are providing service to the non-Jewish population. The Arab population constitutes 12–13% of the total residential care population, lower than the 19–20% in the total population. In residential care, the Arab population is characterized by younger children with severe and profound intellectual disability. The informal family support system is still a very important factor in the Arab family in Israel, a fact that we believe should be strengthened by implementing the British and Danish model of nurse home visitation.

## INTRODUCTION

Israeli Arab society is essentially a traditional society. As such, Israeli Arab society possesses a clear and well-defined system of mores and customs. This system directs and regulates personal and intergroup behavior and relations within society.

In practice, one can distinguish set behavior patterns and even on frequent occasions predict how the individual will behave in a given situation. We are confronting a society where the individual in a significant manner is "directed by factors", whose origins owe more to tradition and values than to environmental conditions, such as poverty or educational neglect, or from conditions of abundance characteristic of a modern society.

The Arabs in Israel constitute close to 20% of the general population[1]. They are considered a traditional population, undergoing rapid processes of change, transition, and modernization. These processes find expression in a rise of the educational level for men and women, improvement in the level of health services, an improvement in the general standard, and constant exposure and ties with the modern Jewish population in Israel.

The Arabs in Israel are subdivided into three ethnic subgroups, in accordance with religious affiliation: Muslims 77%; Christians 14%, and Druze 9%[1]. This division is of particular significance to our subject as religious beliefs are of considerable importance in shaping approaches towards exceptions.

This paper will look at the changes in recent years in the Arab community in Israel towards children with intellectual and developmental disability through research conducted.

## RELIGIOUS ATTITUDES TOWARDS THE EXCEPTIONAL

Professional literature dealing with the subject of stances towards the impaired and exceptional emphasizes the idea that people with physical impairments belong to a social status approximating the status of minority groups[2]. Social discrimination towards them exists and finds expression in negative positions and in an unwillingness to accept them as equals[2]. The cultural variable is of special importance in Israeli society, both because Israel is a society of immigrants, which absorbs Jewish immigration from various cultures, and because it embraces minority groups of various nationalities and religions. The majority groups in Israel are characterized by a pluralism of the values, which are influenced by the culture of the various countries of origin. However, one can assume that the common structure of these values is the traditional Jewish approach, which regards impairment as a punishment leading towards purification and preparation for the world to come and as an attempt to examine the faith of that person[2].

As was noted, existing alongside Jewish Society, non-Jewish minority groups also exist who are still influenced to a large extent by the norms, principles, and cultural values that characterize Arab tradition[3]. The manner in which Arab society in Israel addresses the impaired is also not uniform. There are those who would view impairment as a punishment from heaven for a particular transgression, while others would view it as an arbitrary result of divine will, and some would connect it with "the evil eye", the results of an essentially inexplicable external factor. The Islamic religion views people who are weak both physically and mentally, who do not contribute to society the way a healthy person would, as impaired and therefore one should not place the same demands on them as on healthy individuals. Therefore Islamic religion enjoins an attitude of mercy towards the impaired. It is plausible to assume that the sometimes fatalist approach coupled with popular norms prevalent amongst Israeli Arabs creates a negative attitude towards the impaired[2], which in fact is not founded in Islam.

We encounter a similar approach in Christianity according to which impairment is determined by God, but it is visited as a punishment for transgressions and guilt[4]. They view the impaired as the personification of Jesus, who by his suffering assumed on his shoulders the sins and travails of mankind. Impairment can be viewed as a test to which the impaired and those surrounding him are subjected. In Christian belief, man is called on to withstand negative forces, which attempt to seduce him with disbelief, acquisitiveness, envy, and physical addiction. Man is expected to withstand such temptations. If he should fail to withstand them, he may be punished, and these punishments can find expression in human suffering, illness, and impairment. During the course of history throughout the Christian World, we encounter various approaches to impairment, including positions of pity and mercy contrasted with positions of rejection and lack of acceptance of the impaired[4].

Druze religious beliefs emphasize life after death and the transmigration of souls. According to this belief, life on earth is but a transient status for a person and in his future incarnation he can be in any totally different status or condition and can enjoy a better life. On the other hand, any person can be an invalid or infirm, even if in his previous incarnation he was an important and successful individual. This religious belief is apparently tied with the more positive positions, which the Druze express towards their impaired children. The Druze faith is a branch of Islam, which has a strong neo-Platonic component. The essential tenets of the Druze are kept secret. Nonetheless, it is known that the Druze faith emphasizes the concept of new life after death. In this world, people pray and expect to be born again after their death into a better status and enjoy better health. The belief in the transmigration of souls reinforces an optimistic approach to life and a positive approach towards their impaired children.

In summation, one can state that all three religions to which Arabs in Israel belong believe that impairment is subject to God and therefore one should accept this verdict and the person who suffers from the impairment. Religious faith is not the sole factor determining approaches towards the impaired. Other factors exerting a reciprocal relation with religion are the level of education and the intensity of modernization processes, which the individual in Arab society is experiencing.

## PARENTAL REACTION TOWARDS THE EXCEPTIONAL

The Israeli Arab family would seem to fit the universal model for parental reaction to the birth of a child with a disability[5,6,7] with the crisis taking the following forms:

- The first and the most difficult stage is the crisis that follows a diagnosis of intellectual disability made immediately after birth or in the neonatal period, while the parents are in a state of acute sensitivity. The moment they are apprised that their child is disabled, all their hopes are dashed and their reactions can be traumatic. At this stage, first and foremost, the parents require the attentive ear of a professional person who will be capable of absorbing the initial expressions of disappointment and bitterness. This stage can be called the **change crisis**, the period where the whole world of the parents turns upside down.
- The second stage is described as an **ethical or ideological crisis**, where the parents fear being seen with their disabled child and confronting both family and strangers with the fact. They feel pangs of guilt, personal failure, and apprehension for the future of the child, who will be incapable of independence.
- The third stage is the **reality crisis**. The parents encounter day-to-day problems implicit in the care for the disabled child, difficulties in locating special care, education frameworks, and the struggle to cope with the medical problems, investigations, and treatments. At this stage, parents need practical assistance to deal with real problems such as housework, help with child care, and finding a suitable daily routine.

Recent years have seen a research interest in long-term coping with the stressful situation of having a child with intellectual disability[8]. In this study[8] from the U.S., the personal burden of both younger and older caregivers was examined and it was found that younger caregivers (meaning young parents with young children) reported significantly more unmet service needs, were more apt to mobilize their families to receive help, while older caregivers were more likely to seek spiritual support and had a significantly less personal burden.

## A STUDY OF STRESS, SOCIAL SUPPORT, AND WELL BEING OF ARAB MOTHERS IN NORTHERN ISRAEL

A recent study from the University of Haifa[9] looked at the influence of the social support system of 100 Israeli Arab mothers with intellectually disabled children. In the Nazereth area, 51 mothers were served by the welfare services, while 49 rarely received formal service; 40 mothers lived in an urban area, while 60 lived in rural areas. Of the 100 mothers, 51 agreed to participate in a personal interview (in Arabic by a social worker) in their home, which collected demographic data, the emotional stress perception scale, the structure of psychological stress and well being in the general population, and the quantitative social support scale developed by the authors[9].

The demographic characteristics of the groups (receiving formal support, not receiving, urban and rural) were not significantly different. Marital and economic stress were associated with the amount of social support the mother received, so that mothers with higher informal support had lower marital and economic pressure, whereas formal support had no significant association with the level of stress. It was also found that the marital and parental stress of the mothers who got formal support was higher than that of mothers not asking for help. Concerning well being, the mothers with a broad informal social support reported a greater sense of well being and there was no difference in well being between the mothers using formal support or the ones who rarely used that service.

A significant difference was found in the use of formal services according to the education level and type of residence indicating that urban mothers with lower education used formal services more than rural mothers with higher education.

This study disproved the hypothesis that mothers who use formal support or government services will have a lower level of stress, but instead found that Arab mothers of children with intellectual disability in northern Israel have better use of their informal support system to reduce marital stress, parental stress, economic stress, and have a better well being[9]. This study confirms the experience of home visitation from the U.K., Denmark, and the U.S.[10], where a nurse health visitor will come home as soon as pregnancy is established and follow the child for the first year or more in order to support the natural environment of the family and help the mother establish her informal network, parental education, and a bridge to the service system.

## EXPERIENCE WITH THE BEDOUIN POPULATION IN THE SOUTH OF ISRAEL

The Bedouin population, a minority within the Arab minority, currently numbers 170,000 persons, with 110,000 in the Negev, 10,000 in the central region, and 50,000 in the north of Israel. Most of the Bedouin tribes in the Negev come from the Hejaz, a region in the north of the Arabian penninsula. At present, there are more than 30 elementary schools, 3 high schools, and 3 vocational schools for the Bedouin community in the Negev. All the Bedouin high schools and 60% of the elementary schools in the Negev are located in the seven Bedouin towns. There are clinics in all seven Bedouin towns in the Negev with medical staff including Jews and Arabs. Most of the Bedouin living outside the towns are able to reach the clinics and several mobile clinics provide services in more outlying areas.

The Zusman Child Development Center (CDC) of the Pediatric Division at the Soroka University Medical Center in Beer-Sheva provides health and rehabilitation services to a very large and heterogeneous catch-up population serving most of the south of Israel. The Soroka University Medical Center has 13,000 newborns every year with close to 60% Bedouins, even though they only constitute 20% of the population in the south. Most children with any developmental problem in the south region are referred to the CDC[11,12].

Every year, a total of about 750 new children are referred to the CDC for assessment and treatment by the medical and paramedical staff. About 50% need treatment, 30% need follow-up, and 20% are discharged without need for further intervention. After diagnosis, the therapist and the parents plan an integrative program with special neurological and developmental components suitable for the child, the family, and the rehabilitation services in the local community where the child lives. The rehabilitation therapy is given two to four times a month. The frequency of treatments and follow-up depends on the severity of the disability, the capacity of the family to come to the therapist, and the possibilities for improvement[11,12]. The therapist plans the treatment at home and, during the session, the parents receive instructions connected to the development of the child with a view to capacities and limitations.

One study[11] looked at the compliance with home rehabilitation therapy among 193 families with children with special needs. Lower education level, lower economic level, and being Bedouin corresponded with lower parental compliance. When an intervention program with telephone contact, translation facilities, and detailed explanations was implemented, the compliance rate rose to 76%.

Another study from the same center[12] looked at the utilization and the number of appointments for rehabilitation services by the Bedouin population compared to the Jewish population in the south of Israel. The records of appointments to the CDC between the years 1995–1999 inclusive were studied and the daily records of nine therapists were randomly chosen, three from each discipline (occupational therapy [OT], physical therapy [PT], and speech and language therapy [SLT]) and for four particular months (January, April, July, and October). There were 8,504 appointments during these 4 months of the years 1995–1999, 2,255 of which were for Bedouin and 6,249 for Jewish children. Noncompliance with therapy appointments (NCTA) for the same period for both the Bedouins (31%) and Jewish children (26%), with a significant difference between the two populations, was noted. Of all the Jewish childrens' appointments, the percentage of all three services was similar: 33% to PT, 38% to OT, and 29% to SLT, but for the Bedouin children the percentage between the three services was significantly different: 62% to PT, 34% to OT, and 3% to SLT. These results seem to indicate that the Bedouin families prefer the PT and OT over the SLT. The results of this study again showed the need for planning a model for supplying health services adapted to clients who come from different cultures. According to this model, we need to take in consideration the cultural differences, the accessibility to rehabilitation services, and the economical impact on the family.

## TWO CASE STUDIES

### Case Study 1

Nearly 7-year-old male in a Bedouin family. The father born in 1965, the mother in 1969. He has three healthy brothers and sisters. The father is married to another woman with a healthy son. Both parents without any education. At age 2 years diagnosed with Duchenne Muscular Dystrophy (DMD). The diagnosis was made while he was under ambulatory investigation for failure to thrive and afterwards referred to the CDC for further follow-up and rehabilitation.

After the marriage to the second women (before the diagnosis of DMD was made), the father started to live with the second woman without connection to the first wife and her children. The first wife and the children live about 5 km away from the medical center. She is living on welfare without economic support from the father. He has also not given any emotional support and very rarely he will escort the mother and child to the routine and special examination, including muscle biopsy. Due to this social situation, the condition of the child deteriorated more rapidly and, as an example, orthopedic surgery to elongate the Achilles tendon was postponed several times because the mother could not come alone with the child.

Although the social worker at the CDC tried to intervene and involve the social welfare workers, all efforts failed. Many services to which the child and mother are entitled could not be given to them because the agreement and participation of the father was needed. The child is now 6.5 years old and has never been enrolled in any educational program.

### Case Study 2

Two-year-old male who was born after a normal pregnancy, but even though Down syndrome was diagnosed during pregnancy, the parents decided to continue with the pregnancy. The father was born in 1978, the mother in 1979, both with 12 years of education. The mother is the only wife and they have an older child. This index child was born at term. He was clinically with Down syndrome, suffered from hypoglycemia and polychythemia with congenital heart defect (PDA and PFO). At age 5 days discharged home with developmental and rehabilitation follow-up at the CDC.

At about 1 year, readmitted to the medical center due to failure to thrive, distended abdomen, hepatosplenomegaly, and suspected colitis. Tricuspid regurgitation and pulmonary hypertension were found. Since then (1.5 years), he has stayed hospitalized due to the need for parenteral nutrition and several failed attempts to give oral or tube nutrition.

All this time, at least one and generally both parents are with him. The parents have brought an outside consultant for a second opinion, checked the possibility of transfer to another medical center, but decided to let him stay close to their home. We are not able to determine when, if ever, he can be discharged back home.

## RESIDENTIAL CARE FOR ARAB PERSONS WITH INTELLECTUAL DISABILITY IN ISRAEL

The first residential care center for children with intellectual disability was established in Jerusalem in 1931, and at the time of the establishment of the modern State of Israel in 1948 there was a total of 4 residential care centers providing service to 150 children[13]. The first residential care center for Arabs in Israel was Perak Shalabi in Baka El-Gharbiya established in 1972. Today, out of 57 residential care centers in Israel for persons with intellectual disability, 13 (22.8%) are providing service to the non-Jewish population with the biggest in Kfar Kana (180 residents), established in 1977[14].

For this paper, we looked at the data presently available on the Arab population in residential care in Israel[14]. In Table 1, data are presented on the Arab population in residential care compared with the total population in care. It can be seen that the proportion since 1998 has been constantly 12–13%, which is a little lower than the percentage (19.1%) of Arabs in the total population of Israel in 2002[15]. This lower percentage could be seen as an effect of Arab families keeping their children at home, instead of placement in residential care. In Table 2, the age distribution from residential care centers in 2002 is shown compared with the total population in care and Table 3 has the level of intellectual disability. Again there are differences between the two populations. It can be seen that the Arab population in residential care is characterized as younger and close to 75% with severe or profound intellectual disability. There are very few older Arab persons in residential care. These findings could be interpreted as a result of the burden to the family of a severe or profound child with associated medical problems who then prefers placement in residential care, while the older person with moderate or mild intellectual disability stays with the family at home. A finding we also observed in another study[16] of older persons living at home in Jerusalem, where placement was preferred when the medical burden became too great for the family.

## CONCLUSIONS

In recent years, a change has taken place among Arab families in Israel with intellectually disabled children towards the same norms and values as the Jewish population, where deinstitutionalization and normalization have taken place. The informal family support system is still a very important factor in the Arab family, a fact that we believe should be strengthened by implementing the British and Danish model of nurse home visitation.

**TABLE 1**
**Number of Arab Persons with Intellectual Disability in Residential Care Centers in Israel, Sex Distribution, and Percentage of the Total Population in Care 1998–2002**

| Year | TOTAL | Male | Female | Total Population | % Arabs of Total Population |
|---|---|---|---|---|---|
| 1998 | 749 | 410 | 339 | 6,022 | 12.44 |
| 1999 | 775 | 426 | 349 | 6,122 | 12.66 |
| 2000 | 795 | 438 | 357 | 6,213 | 12.80 |
| 2001 | 820 | 455 | 365 | 6,370 | 12.87 |
| 2002 | 840 | 479 | 361 | 6,352 | 13.22 |

**TABLE 2**
**Age Distribution of Arab Persons with Intellectual Disability in Residential Care Centers in Israel Compared with Total Population in Care in 2002**

| | Age (Years) | | | | | | |
|---|---|---|---|---|---|---|---|
| | 0–9 | 10–19 | 20–39 | 40–49 | 50–59 | 60+ | Total |
| Arabs in 2002 | 96 | 255 | 385 | 77 | 22 | 5 | 840 |
| % | 11.43 | 30.36 | 45.83 | 9.16 | 2.62 | 0.60 | 100.00 |
| Total population in care | 169 | 847 | 2,667 | 1,412 | 899 | 358 | 6,352 |
| % | 2.66 | 13.33 | 41.99 | 22.23 | 14.15 | 5.64 | 100.00 |

**TABLE 3**
**Level of Intellectual Disability of Arab Persons with Intellectual Disability in Residential Care Centers in Israel Compared with Total Population in Care in 2002**

| | Mild | Moderate | Severe | Profound | Other | Total |
|---|---|---|---|---|---|---|
| Arabs in 2002 | 23 | 189 | 421 | 206 | 1 | 840 |
| % | 2.74 | 22.50 | 50.12 | 24.52 | 0.12 | 100.00 |
| Total population in care | 469 | 2,660 | 2,273 | 929 | 21 | 6,352 |
| % | 7.38 | 41.88 | 35.78 | 14.63 | 0.33 | 100.00 |

## BIOSKETCHES

**Isack Kandel**, **MA, PhD,** is Senior Lecturer at the Faculty of Social Sciences, Department of Behavioral Sciences, the Academic College of Judea and Samaria, Ariel. During the period 1985–93, he served as the Director of the Division for Mental Retardation, Ministry of Social Affairs, Jerusalem, Israel. E-mail: Kandeli@aquanet.co.il

**Mohammed Morad, MD**, is Specialist in Family Medicine, Lecturer in Family Medicine at the National Institute of Child Health and Human Development, Division of Community Health, Ben Gurion University of the Negev and the Medical Director of a large area clinic in the city of Beer-Sheva. He has publications on Bedouin health, health aspects, spiritual health, and aging in persons with intellectual disability, and is a presenter on topics such as health policy and services for the disadvantaged at national and international conferences. E-mail: morad62@barak-online.net

**Gideon Vardi, MD, MPH**, is a specialist in pediatrics, child health and development working at the Zusman Child Development Center, Division of Pediatrics, Faculty of Health Sciences, Ben Gurion University of the Negev in Beer-Sheva, Israel. His interest and several publications are in the field of child health and human development, rehabilitation, intellectual disability and disability. E-mail: GideonVa@clalit.org.il

**Joseph Press, MD**, is Professor of Pediatrics with interest in emergency medicine and rheumatology. He serves as the director of the Pediatric Emergency Department and chairman of the Pediatric Division, Soroka University Medical Center, Faculty of Health Sciences, Ben Gurion University of the Negevin

Beer-Sheva and the prime mover behind the construction of the Saban Children's Medical Center at Soroka. E-mail: press@bgumail.bgu.ac.il

**Joav Merrick, MD, DMSc,** is Professor of Child Health and Human Development affiliated with the Zusman Child Development Center, Division of Pediatrics and Community Health at the Ben Gurion University, Beer-Sheva, Israel; the Medical Director of the Division for Mental Retardation, Ministry of Social Affairs, Jerusalem; and the Founder and Director of the National Institute of Child Health and Human Development. He has numerous publications in the field of child and human development, rehabilitation, intellectual disability, disability, health, welfare, abuse, advocacy, quality of life, and prevention. Dr. Merrick received the Peter Sabroe Child Award for outstanding work on behalf of Danish Children in 1985 and the International LEGO-Prize ("The Children's Nobel Prize") for an extraordinary contribution towards improvement in child welfare and well being in 1987. E-mail: jmerrick@internet-zahav.net. Website: www.nichd-israel.com
